# Signaling output: it's all about timing and feedbacks

**DOI:** 10.15252/msb.20156642

**Published:** 2015-11-30

**Authors:** Nils Blüthgen

**Affiliations:** ^1^ Charite Universitätsmedizin BerlinBerlinGermany

**Keywords:** Quantitative Biology & Dynamical Systems, Signal Transduction, Development & Differentiation

## Abstract

The central questions in understanding signaling pathway specificity are how these pathways encode which stimulus is present and how this stimulus is decoded to yield the correct cell fate decision. In their recent work, Ryu *et al* (2015) show by stimulation experiments with different ligands how the differential engagement of feedback and feed‐forward regulation leads to different dynamics of pathway activity, which in turn alters cell fate. Moreover, they show that by considering the timescales of the feedback regulations, the different cellular responses can be triggered with pulsed stimulations by a single ligand.

Cells respond to a large number of stimuli by employing only a small number of intracellular signaling pathways, and as such, they need to reuse the same pathway to transmit very different signals. This in turn leads to the phenomenon that the activation of the same signaling pathway results in different—and often even opposing—cell fates. Differentiation of PC12 cells serves as the paradigm model system to study how one signaling pathway can control different cell fates (Marshall, 1995). If PC12 cells are stimulated with neural growth factor (NGF), they show prolonged activation of the ERK signaling pathway and start to differentiate. In contrast, stimulation with epidermal growth factor (EGF) triggers a short pronounced pulse of ERK activity, leading to cell proliferation (Fig 1A). It is known that cells can decode and interpret the length of ERK signaling: Short‐term ERK signaling induces cFOS and FRA1 transcription, but the resulting proteins are very unstable unless phosphorylated by ERK, and therefore, only long‐term ERK signaling can lead to sufficient protein expression (Murphy *et al*, 2002, 2004).

**Figure 1:**
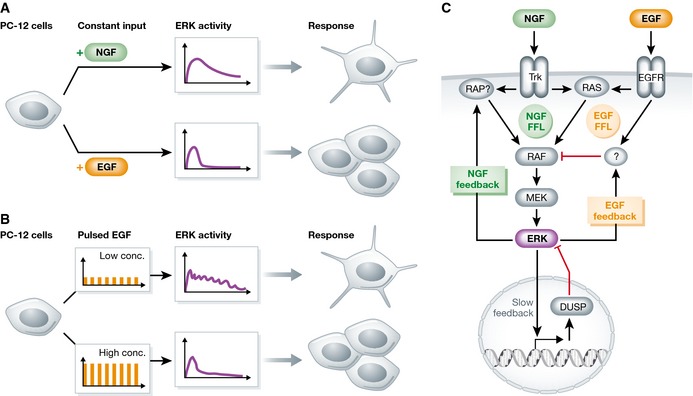
Response and wiring of the EGF/NGF network (A) When PC‐12 cells are stimulated with NGF, cells show long ERK activity and differentiate. When they are stimulated with EGF, they show a short peak of ERK activity and proliferate. (B) Stimulation of PC‐12 cells with short pulses (3 min) with low EGF concentration results in prolonged ERK activity, similar to NGF treatment, and subsequent differentiation. Pulses with higher EGF concentration lead to a short response, followed by proliferation. (C) The proposed network topology includes an EGF‐dependent negative feedback, an NGF‐dependent positive feedback, intertwined with stimulus‐dependent feed‐forward loops (FFL). Long‐term desensitization occurs via transcriptional induction of phosphatases (DUSP proteins).

Twenty years after the observation that duration of ERK signaling encodes cell fates (Marshall, 1995), Ryu *et al* (2015) now set out to analyze the properties of the signaling network that can give rise to prolonged versus short signaling. Their approach was heavily inspired by how engineers would probe an electrical circuit: They stimulated the system using pulses with different frequency and amplitude and monitored signaling dynamics. Using microfluidics, the authors challenged PC12 cells with defined input pulses of EGF and NGF and monitored ERK pathway activity in real time in single cells using FRET reporters. With this setup, they were able to essentially probe how long cells signal when they are treated with different doses of EGF and NGF, and how quickly cells can be re‐stimulated. When cells received constant levels of EGF and NGF, dosage did not matter, and EGF led to short‐term pathway activity, whereas NGF led to long‐term pathway activity in most cells, essentially confirming previous results (Marshall, 1995). The responses to the pulses of growth factors however (as illustrated in Fig 1B) are strikingly different and reveal essential features of the network. Specifically, when cells are stimulated with repeated pulses of 3 min, low concentrations of EGF led to prolonged ERK activation, whereas high concentrations of EGF led to short ERK activity. From this data, a lot can be learned about how the network is wired. For instance, since the initial response of ERK to EGF is identical for low and high doses, a negative feedback alone cannot explain why pulses of low doses lead to prolonged signaling, and the authors reasoned that in addition to feedback regulation, feed‐forward signaling is required.

The advantage of live‐cell imaging is that one can also assess individual differences within a clonal cell population. An interesting aspect of these data is that the response to NGF treatment is extremely heterogeneous: Some cells show long ERK activity, while others show only a short response. Correlation analysis shows that long pathway activation occurs when the first peak of ERK activity is high. The authors use this information to complete their network model: A plausible network motif that could cause this behavior is a thresholded positive feedback, which causes prolonged activation only when ERK exceeds a certain activation level. A further hint toward the existence of positive feedback regulation comes from short‐term stimulation experiments: When cells are stimulated for 10 min with NGF, again a subpopulation of cells show a high peak of ERK activity, followed by long ERK activation even though the ligand is no longer present.

The authors build a model of feedback and feed‐forward regulation (Fig 1C), extending previous work by Santos *et al* (2007), who reconstructed the network using a reverse engineering approach and data from siRNA perturbations. In their updated model, fast feedbacks and feed‐forward loops are integrated at the level of RAF, a protein with dozens of phosphorylation sites and a known feedback target of ERK, but it remains open which molecules are engaged in these positive and negative feedbacks and feed‐forward loops. Earlier work showed that while both NGF and EGF activate RAS, NGF additionally signals through RAP, suggesting that the RAP pathway could be the feed‐forward loop in the NGF branch (Sasagawa *et al*, 2005). An additional slow negative feedback is responsible for desensitization on a long timescale. In their model, this occurs by the induction of dual‐specificity phosphatases, a class of transcriptional feedback regulators of ERK.

Taken together, the idea to challenge networks with very defined input patterns with different frequencies and amplitudes is extremely potent to uncover the timescales of the major network components shaping the signal. Such an approach unveils the existence and properties of feedback and feed‐forward loops. With emerging technologies like microfluidics and optogenetics (Aoki *et al*, 2013) it becomes feasible to challenge networks in a defined way. In addition, fast fluorescent readouts make it possible to study pathway activity in real time in many systems. The arena is open to use these approaches to unravel how different network features like feedbacks and feed‐forward loops in signaling interact to encode specific signals.
